# A novel circular RNA circPPFIA1 promotes laryngeal squamous cell carcinoma progression through sponging miR-340-3p and regulating ELK1 expression

**DOI:** 10.1080/21655979.2021.1959866

**Published:** 2021-08-30

**Authors:** Yu Shuang, Jing Liu, Juntao Niu, Wenyu Guo, Chao Li

**Affiliations:** Department of Otorhinolaryngology Head and Neck Surgery, The Second Hospital of Tianjin Medical University, Tianjin, P.R. China

**Keywords:** Circppfia1, lscc, miR-340-3p, elk1, ceRNA

## Abstract

Abnormal expression of circular RNA (circRNA) is closely related to the occurrence and development of many cancers. By screening the expression of circRNA, we identified a novel circRNA termed as has_circ_0023326 in laryngeal squamous cell carcinoma (LSCC). We verified the expression of circPPFIA1 and found that it was upregulated in LSCC tissues compared to the adjacent normal tissues. Functional studies were carried out to detect the effect of circPPFIA1 expression on the phenotype of LSCC cells. These results suggest that circPPFIA1 knockdown can suppress the proliferation, migration, and invasion of LSCC cells, while circPPFIA1 overexpression can promote these processes. Mechanistically, miR-340-3p was predicted to be the target miRNA sponged by circPPFIA1 as confirmed through the luciferase assay and rescue experiments. In addition, miR-340-3p was found to target ELK1 and inhibit its expression. Taken together, circPPFIA1 promotes the progression of LSCC via the miR-340-3p/ELK1 signaling axis, which may serve as a novel prognostic or therapeutic target for LSCC.

## Introduction

Laryngeal cancer is a common primary malignant tumor of the head and neck, accounting for 5.5%-5.6% of all tumors [1,2]. Laryngeal squamous cell carcinoma (LSCC) is the most common type of laryngeal cancer, which is highly invasive and accounts for approximately 95% of laryngeal cancer cases [3,4]. Moreover, almost 60% of patients with LSCC already have advanced disease, and the current therapies available for LSCC are still unfavorable due to their limitations, and these patients still face the risk of tumor recurrence, metastasis, and drug resistance [5,6]. Thus, it is urgent to expand our understanding of the molecular mechanisms involved in LSCC development.

CircRNA may play a role in regulating tumor development, including laryngeal squamous carcinoma, by acting as a sponge for related miRNAs [7]. Studies have found that the abnormal expression of multiple miRNAs in laryngeal squamous carcinoma tissues is closely related to the occurrence and development of laryngeal squamous carcinoma [8]. Wu et al. found that circCORO1C can promote the development of LSCC through the let-7 c-5p/PBX3 signaling axis [9]. CircRNA_100290 also plays an oncogenic role in LSCC by sponging miR-136-5p and regulating the expression of RAP2C [10]. In addition, circ-LDLRAD3 has been shown to be closely related to the development and prognosis of laryngeal squamous carcinoma [11].

MicroRNAs (miRNAs) are single-stranded non-coding RNA molecules, ranging from 19 to 25 nucleotides in length, and are encoded by endogenous genes [12]. miRNAs play an important regulatory role in human pathophysiology and are involved in signal activation, cell proliferation, differentiation, and death by regulating the expression of signaling molecules [13]. Previous studies have shown that miR-613 inhibits the development of LSCC by targeting PDK1 [14]. miR-506 plays an anti-cancer role in LSCC by targeting YAP1 [15]. MiR-154 attenuates LSCC progression by negatively regulating the expression of GALNT7 [16].

In the present study, we identified a novel circular RNA (circPPFIA1) in LSCC cells. CircPPFIA1 expression is upregulated in LSCC. We hypothesis that circPPFIA1 must play critical roles in the LSCC progression. Here, the aim and goal of this study was to elucidate the biological function of circPPFIA1 in LSCC development and the underlying mechanism.

## Methods and materials

### Clinical samples

A total of 30 patients with LSCC who underwent surgery at the No. 2 Hospital of Tianjin Medical University were included in this study. Adjacent normal tissues were taken 5 cm away from the edge of the tumor. The diagnosis of LSCC was confirmed via histological examination. Patients with LSCC who had received prior anti-tumor treatment or a history of other solid tumors were excluded from this study. This study was approved by the Human Research Ethics Committee of the No. 2 Hospital of Tianjin Medical University. Informed consent was obtained from all patients.

### Cell culture

The human bronchial epithelial cell line NHBEC was acquired from the Chinese Academy of Sciences (Shanghai, China) and was grown in bronchial epithelial cell growth medium containing 10% FBS (Gibco, USA). The LSCC cell lines TU212, TU177, LSC-1, TU686, and AMC-HN-8, were also obtained from the Chinese Academy of Sciences and were cultured in RPMI 1640 medium (Gibco, USA) containing 10% fetal bovine serum (FBS, Gibco), 100 U/mL penicillin and 100 mg/mL streptomycin (Gibco, MA, USA). All cells were cultured in an atmosphere of 5% CO_2_ at 37°C [17].

### Cell transfection

A short hairpin RNA (shRNA) of circPPFIA1 and ELK1, as well as their negative controls, were synthesized by GenePharma Co., Ltd (Shanghai, China). Full-length circPPFIA1 was cloned into PLCDH-ciR plasmids (Biovector, Beijing, China) to construct a circPPFIA1-expressing vector, and an empty plasmid was used as a negative control. The oligonucleotides miR-340-3p mimic, miR-340-3p inhibitor, mimic normal control (NC), and inhibitor NC were synthesized by Ribobio (Guangzhou, China). Cell transfection was performed using Lipofectamine™ 2000 reagent (Invitrogen, USA) according to the manufacturer’s recommendations.

### Methlthiazoletrazolium (MTT) assay

The transfected cells were cultured in an incubator until the cells were in monolayer covered with a hole bottom (96-hole flat plate). An MTT solution (10 μL, 5 mg/mL, 0.5% MTT) was added to each well, and the culture was continued for another 4 h. After removing the supernatant, 150 µL dimethyl sulfoxide (DMSO) was added to each well of the remaining cells, and the crystals were set to oscillate at a low speed for 10 min to fully dissolve. The absorbance of each well was measured at the wavelength of 490 nm [18].

### Transwell assay

The Transwell assay was performed to determine the invasion and migration abilities of the treated LSCC cells. After 48 h of transfection, Transwell cell culture inserts (8 mm pore size; Falcon; BD Biosciences) were placed into 48-well plates to generate upper and lower chambers. The membrane was hydrated with FBS 2 h prior to use. Then, we used Matrigel (BD Biosciences, San Jose, CA) to coat the upper part of the membrane. Afterward, we cultured the cells for 1 h at 37°C for gelation. RPMI-1640 (600 µL) with 10% FBS and 1 × 10^5^ cells/well were placed into the lower and upper chambers, respectively. After 24 h of culture, the number of invading cells was counted under a microscope [18].

### Colony formation assay

The cells of each experimental group at the logarithmic growth phase were digested with trypsin, and cell suspensions were prepared by adding complete cell culture medium. The cells were seeded in 6-well culture plates, with each well containing 500–1000 cells. The inoculated cells were shaken well and gently placed in an incubator for further culture. Colony size was observed under a microscope. When the individual clones have grown to a suitable size, 1000 μL of an impurity-free crystal violet dye was added to each well to dye the cells for 2 min. The cells were then photographed [18].

### Quantitative realtime PCR (qPCR)

The total RNA was isolated with a TRIzol kit (Beyotime, Shanghai, China) according to the manufacturer’s instructions. Thereafter, RNA was reversed transcribed into cDNA which was further used as the template in PCR reaction using SYBR Premix Ex Taq (Takara, Dalian, China) on a Light Cycler®480 System (Roche, USA). mRNA and miR levels were normalized to GAPDH and U6 respectively. The relative expression level was calculated using 2-ΔΔCt method [19].

### Western blot

RIPA (Sigma-Aldrich, USA) reagents were used to extract proteins from hAECs. Protein concentration was determined using a bicinchoninic acid (BCA) kit (Sigma-AldrPPROM, USA). The extracted proteins (20 µg/lane) were then separated using a 15% sodium dodecyl sulfate polyacrylamide gel electrophoresis (SDS-PAGE gel), transferred onto polyvinylidene fluoride (PVDF) membranes (Bio-Rad, USA), and blocked with 5% skimmed milk for 2 h. The membranes were then incubated with primary antibodies including ki67 (abcam, 1:1000), ELK1 (abcam, 1:800) and GAPDH (Bioss, 1:2000) at 4°C overnight, followed by incubation with the corresponding goat anti-mouse antibodies and goat anti-rabbit IgG antibodies for 1 h. Subsequently, the membranes were stained with an ECL western blotting kit (ab133406, Abcam, USA). GAPDH was used as a loading control. Finally, the protein bands were visualized using an enhanced chemiluminescence system (Thermo Fisher Scientific, USA) [20].

### RNA pull-down assay

To demonstrate the interaction between circPPFIA1 and miR-340-3p, we performed an RNA pull-down assay. A biotinylated probe was designed to bind to the junction region of circPPF1A1 or miR-340-3p. An oligonucleotide probe was used as a negative control. Approximately 1 × 10^7^ cells were lysed in lysis buffer and incubated with 3 μg biotinylated probe for 2 h. The cell lysates were incubated with streptavidin magnetic beads (Life Technologies, Gaithersburg, MD, USA) for 4 h to pull down the biotin-conjugated RNA complex. The RNA complexes were washed with lysis buffer five times. The bound miRNA in the pull-down complex was extracted using TRIzol reagent and analyzed via RT-qPCR [20].

### Luciferase assay

Wild type (WT) circPPFIA1 or ELK1 and their mutant sequences (mut) were cloned into the luciferase reporter psiCHECK2. TU212 and TU177 cells (4 × 10^4^ cells/well) were cultured overnight in 24-well plates. TU212 and TU177 cells were transfected with the reporter plasmid and a miR-340-3p mimic or negative control using Lipofectamine™ 2000 reagent. A Renilla plasmid was co-transfected as an internal control. After 24 h, luciferase activity was measured using a dual luciferase reporter assay kit (Yeasen, Shanghai, China) [18].

### Xenograft models

Mouse xenograft tumor models were established for *in vivo* studies. TU212 cells transfected with circPPFIA1 shRNA were subcutaneously injected into nude mice. The volumes of the xenograft tumors were measured every week for 6 weeks. At the end of the sixth week, the mice were sacrificed, and the weight of the tumors was measured. The animal study was approved by the ethics committee of Tianjin Medical University [21].

### Immunohistochemical (IHC) analysis

Paraffin-embedded tissue samples were sliced into 4-μm sections. Following deparaffinization, rehydration, and antigen retrieval, the sections were blocked with 5% BSA. The sections were then incubated with primary antibodies against ELK1 and Ki67 (Abcam, Cambridge, USA) at 4°C overnight, followed by incubation with the corresponding secondary antibody at room temperature for 2 h. Subsequently, the slides were rinsed with PBS three times, stained with diaminobenzidine (DAB), and counterstained with hematoxylin. Finally, the slides were photographed under a microscope (Leica, Germany) [19].

### Statistical analysis

GEO2R program was used to analyze the data from GSE117001 database. All assays were carried out in triplicate and all results were shown as the mean ± standard deviation (SD). SPSS 22.0 (SPSS Inc., USA) was employed to conduct statistical analysis. Comparisons between two or more groups were analyzed by Student’s t-test or one-way ANOVA followed by Tukey’s test. P < 0.05 was regarded to be statistical significance.

## Results

Here in the study, we identified a novel circular RNA (circPPFIA1) which was upregulated in LSCC cells. We hypothesis that circPPFIA1 must play critical roles in the LSCC progression. Here, the aim and goal of this study was to elucidate the biological function of circPPFIA1 in LSCC development and the underlying mechanism.

### circPPFIA1 was highly expressed in LSCC

**Figure 1.**
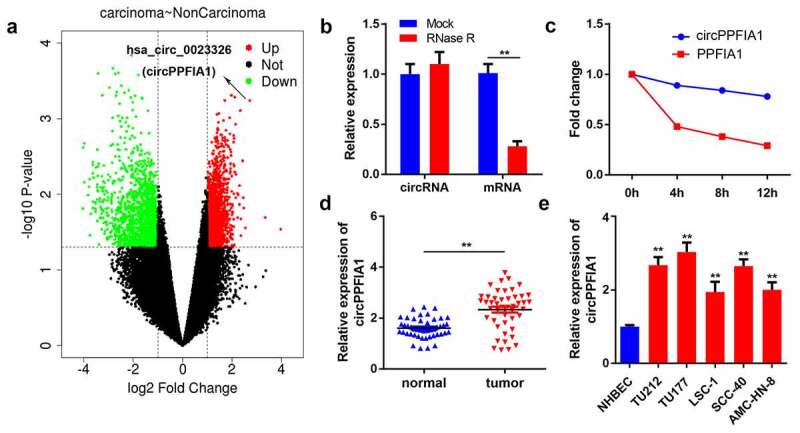
circPPFIA1 expression is upregulated in both LSCC tissues and cells. (a) A screen based on the GEO dataset is shown. (b) after RNase R treatment, RNA expression levels were evaluated via a qPCR assay (n = 3). (c) after actinomycin D treatment for 0, 4, 8, or 12 h, RNA expression levels were evaluated via a qPCR assay (n = 3). (d) circPPFIA1 expression was measured by qPCR in LSCC tissues and the corresponding para-cancerous tissue (n = 30). (e) qPCR was conducted to measure circPPFIA1 expression in LSCC cell lines (TU212, TU177, LSC-1, TU686, and AMC-HN-8), using NHBEC cells as a control (n = 3). **P < 0.01 vs the NHBEC group

We first analyzed the GSE117001 dataset using the R language to screen for dysregulated circRNAs in LSCC and found that has_circ_0023326 was significantly upregulated in LSCC (Figure 1a). circRNAs are much more stable owing to their looped structure. RNase R was used to confirm the stability of circPPFIA1. The results showed that after RNase R treatment, the level of linear PPFIA1 decreased, but the level of circular PPFIA1 did not change (Figure 1b). Subsequently, LSCC cells were treated with actinomycin D, after which the half-lives of circPPFIA1 and linear PPFIA1 were evaluated. The results indicated that circPPFIA1 has a longer half-life than linear PPFIA1 (Figure 1c). These findings indicated the stability of circPPFIA1. We then detected the expression levels of circPPFIA1 using qPCR. The results demonstrated that circPPFIA1 was remarkably upregulated in LSCC tissues and cell lines compared to that in normal tissues and NHBEC cells, respectively (Figure 1(d -e)).

### Knockdown of circPPFIA1 inhibited the proliferation and migration of LSCC cells

**Figure 2.**
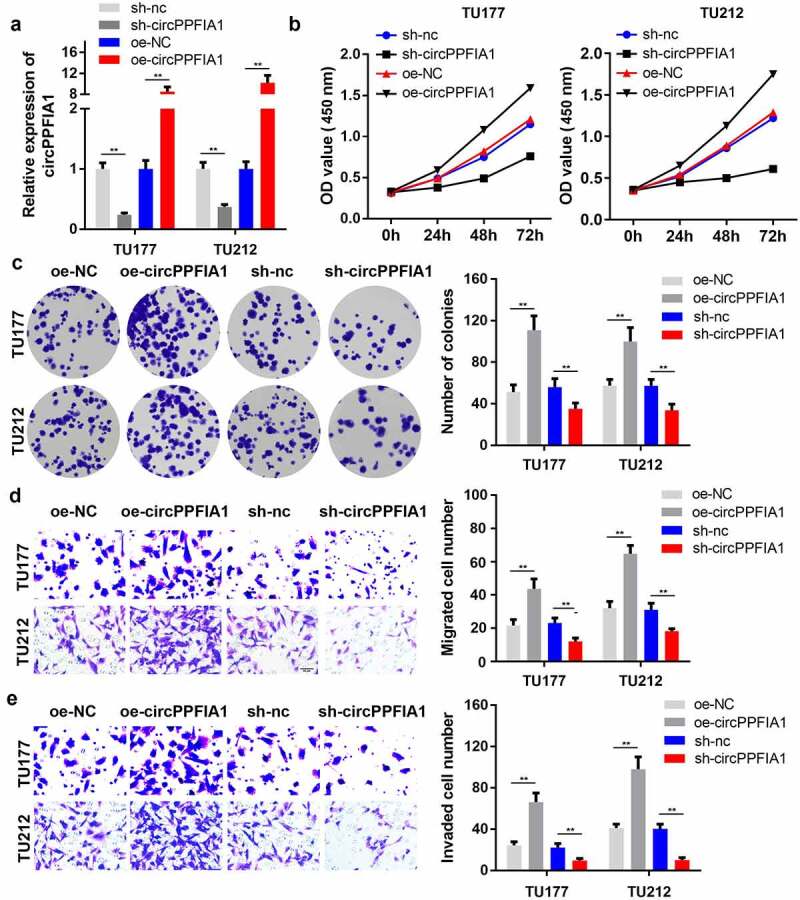
The effects of circPPFIA1 silencing on the proliferation and invasion of LSCC cells. (a) The transfection efficiency of si-circPPFIA1, oc-circPPFIA1 or their negative control in TH177 and TU212 cells was detected via qPCR (n = 3). (b) The proliferation of transfected TH177 and TU212 cells was tested via the MTT assay (n = 6). (c) A colony formation assay was performed to detect the growth of LSCC cells (n = 6). (d, e) Cell migration and invasion ability were analyzed via a matrigel transwell assay using transfected TH177 and TU212 cells (n = 6). **P < 0.01

As circPPFIA1 is highly expressed in LSCC tissues and cell lines, we speculated that circPPFIA1 plays a crucial role in LSCC development. We then established silencing and overexpression vectors of circPPFIA1. qPCR analysis indicated that the shRNA and overexpression plasmid for circPPFIA1 worked well (Figure 2a). Results of the MTT assay indicated that circPPFIA1 silencing suppressed the proliferation of LSCC cells, while the overexpression of circPPFIA1 promoted proliferation (Figure 2b). Colony formation consistently indicated that circPPFIA1 silencing inhibited the formation of LSCC cell colonies, while circPPFIA1 overexpression promoted colony formation (Figure 2c). Transwell assays were then performed to evaluate the migration and invasion abilities of LSCC cells. As the results showed, circPPFIA1 knockdown reduced the number of migrating and invading cells while the overexpression of circPPFIA1 reversed these findings (Figure 2d, Figure 2e).

### circPPFIA1 sponges miR-340-3p in LSCC

**Figure 3.**
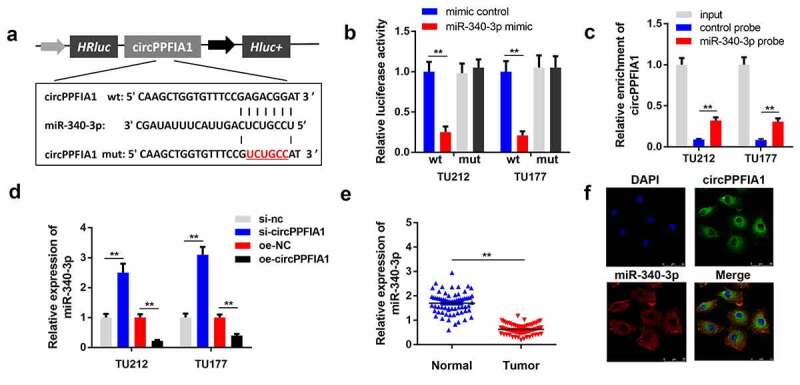
circPPFIA1 knockdown inhibited the growth of LSCC cells *in vivo*. TU212 cells with stably silenced circPPFIA1 and normal TU212 cells were injected into mice to establish an *in vivo* mouse model. (a) images of tumors from the mice of the sh-nc and sh-circPPFIA1 groups are shown (n = 6). (b) the growth curves of the tumors. (c) tumor weights were determined and are shown (n = 6). (d) IHC was performed to detect the expression of Ki67 and SOCS6 in the tumor tissues (n = 6). **P < 0.01 vs the sh-nc group

Numerous studies have reported that circRNAs sponge miRNAs to regulate downstream gene expression and participate in the regulation of cell physiology. We performed bioinformatics analysis to predict the potential miRNA targets of circPPFIA1 and found that miR-340-3p (Figure 3a) is potential target of circPPFIA1. The luciferase activity of the reporter carrying wt-circPPFIA1 was significantly reduced by the miR-340-3p mimic, but not mimic NC. Neither the miR-340-3p mimic nor mimic NC changed the luciferase activity of the reporter carrying mut-circPPFIA1 (Figure 3b). This result confirmed the interaction between circPPFIA1 and miR-340-3p. Results of the RNA pull-down assay indicated that miR-340-3p directly interacts with circPPFIA1 (Figure 3c). qPCR results indicated that circPPFIA1 overexpression reduced the level of miR-340-3p while circPPFIA1 silencing increased it (Figure 3d). Figure 3e demonstrates that miR-340-3p is downregulated in LSCC tissues. FISH analysis indicated that both circPPFIA1 and miR-340-3p were mainly located in the plasma and there was co-localization between circPPFIA1 and miR-340-3p (Figure 3f).

### miR-340-3p targets ELK1 in LSCC cells

**Figure 4.**
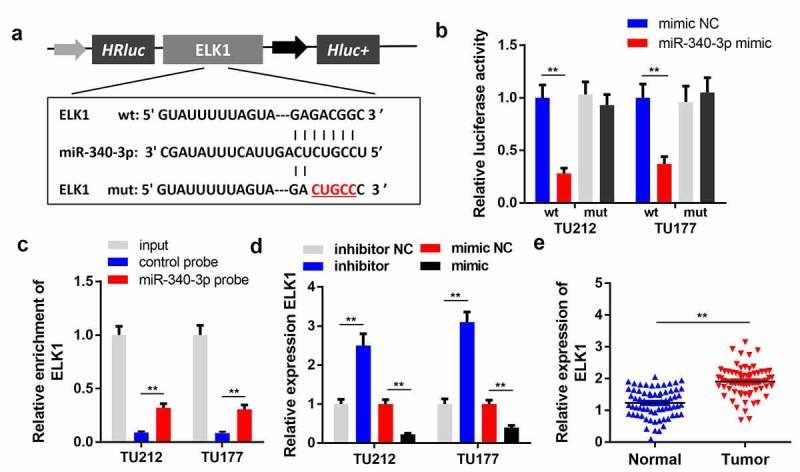
circPPFIA1 acted as a sponge of miR-340-3p. (a) The predicted wt and mut circPPFIA1 binding sites in miR-340-3p. (b) TH177 and TU212 cells were co-transfected with wt circPPFIA1 or mut circPPFIA1 and mimic plasmids. A luciferase assay was performed to detect the binding between circPPFIA1 and miR-340-3p (n = 3). (c) An RNA pull-down assay was performed to further verify the interaction between circPPFIA1 and miR-430-3p (n = 3). (d) qPCR was conducted to evaluate the expression of miR-340-3p (n = 3). (e) miR-340-3p expression levels were measured in 30 pairs of LSCC tissues and adjacent normal tissues (n = 30). (f) A FISH assay was performed to detect the locations of circPPFIA1 and miR-340-3p (n = 3). **P < 0.01

To explore the underlying mechanism of circPPFIA1 in LSCC, we studied the targets of miR-340-3p. Figure 4a shows the binding sites between ELK1 and miR-340-3p. In the luciferase assay, we found that miR-340-3p decreased the luciferase activity of the reporter carrying wildtype ELK1, but not that of the mutant-type reporter (Figure 4b). The RNA pull-down assay results showed that miR-340-3p directly interacted with ELK1 in (Figure 4c). In addition, miR-340-3p significantly inhibited the expression of ELK1, whereas miR-340-3p knockdown promoted ELK1 expression (Figure 4d). qPCR results further indicated that ELK1 was upregulated in LSCC tissues, which further verified our prediction (Figure 4e).

### ELK1 silencing or miR-340-3p overexpression can reverse the function of circPPFIA1 in LSCC

**Figure 5.**
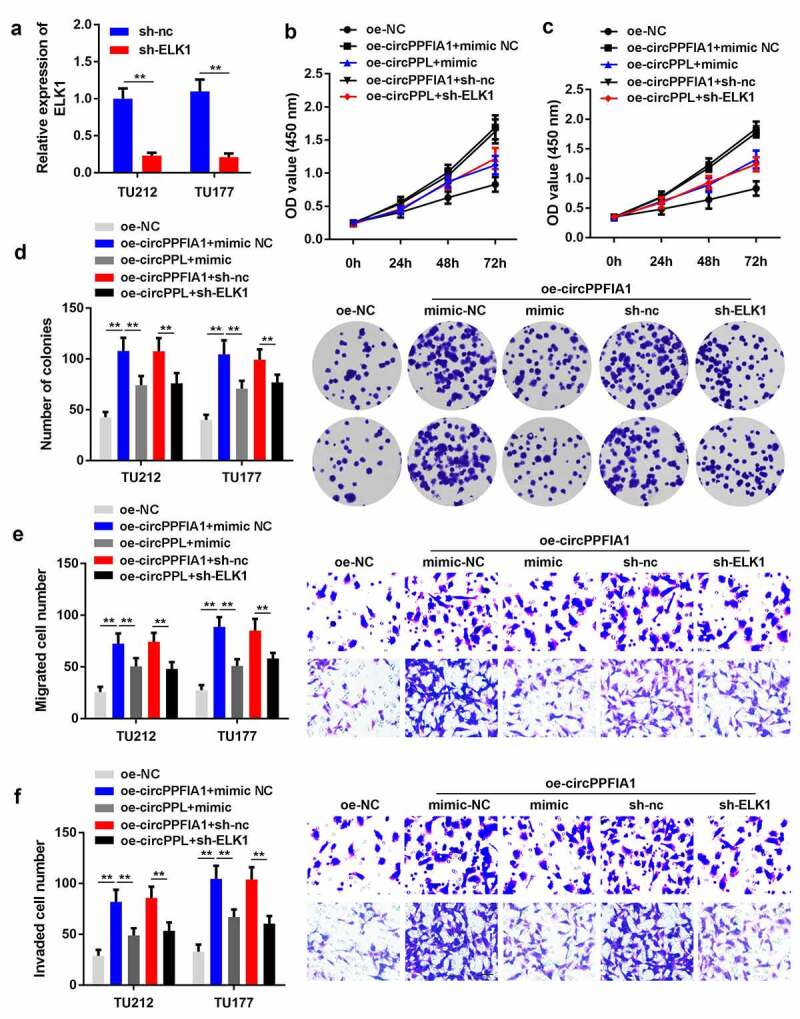
ELK1 is a target of miR-340-3p. (a) the predicted binding site of ELK1 and miR-340-3p is shown. (b) the target relationship between ELK1 and miR-340-3p was confirmed via a dual-luciferase reporter assay (n = 3). (c) an RNA pull-down assay was performed to detect the direct interaction between miR-340-3p and ELK1(n = 3). (d) ELK1 levels were measured via qPCR in miR-340-3p-overexpressing or silenced LSCC cells (n = 3). (D) ELK1 levels were measured in 30 paired normal and tumor tissues via qPCR (n = 30). **P < 0.01

As regulated by circPPFIA1 and miR-340-3p, we predicted that ELK1 also participates in the biological processes of LSCC cells. We co-transfected circPPFIA1-overexpressing (oe-circPPFIA1) and ELK1-silencing (sh-ELK1) vectors, as well as a circPPFIA1-overexpressing vector and a miR-340-3p mimic. First, transfection with the ELK1 silencing vector significantly reduced the expression level of ELK1 (Figure 5a). Co-transfection of oe-circPPFIA1, sh-ELK1, oe-circPPFIA1, and miR-340-3p mimic reversed the effect of circPPFIA1 overexpression on cell proliferation (Figure 5(b-c)), colony formation (Figure 5d), migration (Figure 5e), and invasion (Figure 5f).

### circPPFIA1 accelerated the growth of LSCC cells

**Figure 6.**
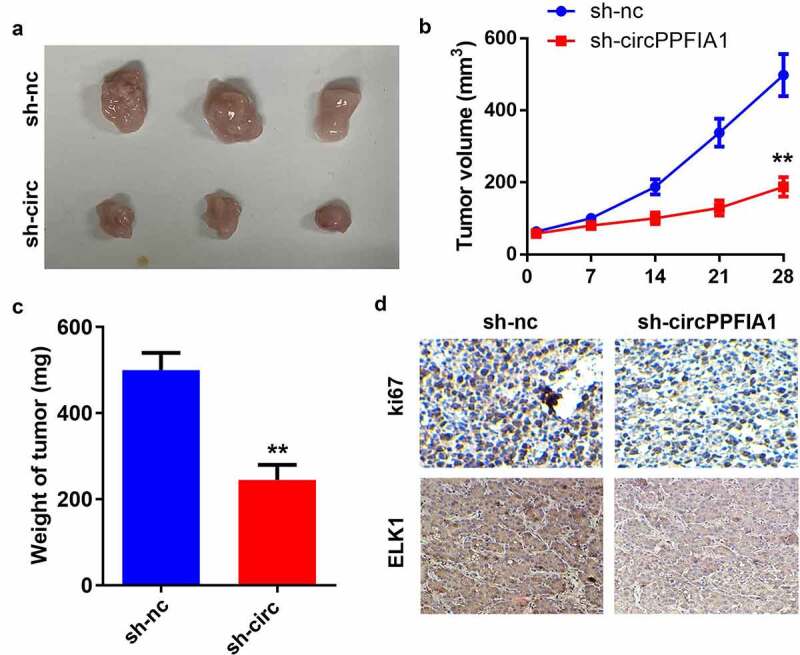
miR-340-3p suppressed the biological functions of LSCC by targeting ELK1. (a) transfection efficiency was measured via qPCR in transfected TH177 and TU212 cells (n = 3). (b, c) cell proliferation was measured via the MTT assay (n = 6). (d) Colony formation was determined, and the number of colonies was quantified (n = 6). (e) cell migration and invasion capability was measured using the Transwell assay (n = 6). **P < 0.01

Next, we carried out an animal study to further investigate the biological effects of circPPFIA1 *in vivo*. A stable TU212 cell line was established via transfection of sh-circPPFIA1 and its control vector. (Figure 6(a-b)) show that tumor growth was inhibited through the knockdown of circPPFIA1. The weight of the tumors was reduced after sh-circPPFIA1 treatment (Figure 6c). IHC staining of Ki67 confirmed that circPPFIA1 knockdown inhibited Ki67 expression in the tumor tissues. Moreover, circPPFIA1 knockdown also inhibited the expression of ELK1 in tumor tissues, which further confirms the interaction between circPPFIA1 and ELK1 (Figure 6d).

## Discussion

LSCC is the most common type of laryngeal cancer, which is highly invasive. It is urgent to expand our understanding of the molecular mechanisms involved in LSCC development. Increasing evidences have confirmed the critical role of circRNAs in regulating tumor development. Thus, we tried to explore the role of circRNAs in the progression of LSCC. In the present study, we identified a novel circRNA, circPPFIA1. The stability of circPPFIA1 was confirmed through its stable expression under RNase R digestion and actinomycin D treatment. The dysregulated expression and biological function of circPPFIA1 were first investigated in this study. The upregulation and stability of circPPFIA1 make it an ideal biomarker for the diagnosis or treatment of certain diseases. Here, circPPFIA1 has been found to play an oncogenic role in LSCC development.

We then attempted to elucidate the molecular mechanism of circPPFIA1. circRNAs can sponge miRNAs to block their inhibitory function against their target genes. Numerous miRNAs have been shown to participate in the biological processes surrounding LSCC. For instance, miR-613 attenuates LSCC development by modulating PDK1 [22]. miR-936 inhibits the proliferation, migration, and invasion of LSCC cells by targeting GPR78 [23]. MiR-194 acts as a tumor suppressor in LSCC by inhibiting Wee1 expression [24], and miR-340-3p has been demonstrated to play critical roles in LSCC progression. miR-340 could significantly attenuate Hep-2 cell-derived tumor growth through the EZH2/p27 signaling pathway *in vitro* and *in vivo* [25]. It can also target YAP1 to mediate the effect of SNHG3 on LSCC progression. We found that miR-340-3p was downregulated in LSCC, which was consistent with the results of previous studies. The rescue experiments we performed further confirmed the interaction between circPPFIA1 and miR-340-3p. Hence, we confirmed the anti-cancer effect of miR-340-3p in LSCC.

To further elucidate the underlying mechanism for this, we predicted the target genes of miR-340-3p and focused on ELK1 as a potential target gene. ELK1 is a transcription factor that can be activated by the MAPK/ERK pathway, further activating the transcription of downstream genes such as c-Fos, cancerous inhibitor of protein phosphatase 2A (CIP2A) [26], and the E3 ubiquitin ligase PARK2 [27], and can also interact with other proteins such as thyroid transcription factor FOXE1 or aPKC-iota to co-regulate TERT and aPKC expression, respectively [28,29]. ELK1 plays a crucial role in the development of human cancers. The suppression of Elk1 inhibits thyroid cancer progression by mediating PTEN expression [30]. ELK1 induced TRPM2-AS and enhances the growth of gastric cancer cells via miR-195/HMGA1 signaling [31]. However, the role of ELK1 in LSCC remains unclear. We demonstrated that ELK1 is upregulated in LSCC and acts as a target gene of miR-340-3p as well as a ceRNA of circPPFIA1. ELK1 has been reported to be targeted by miR-30-5p, miR-185-5p, and miR-326 [32–34], but this is the first study wherein ELK1 was proven to be a target of miR-340-3p. However, we did not determine the downstream genes activated by ELK1. More studies should be carried out to elucidate the precise mechanisms regarding this.

## Conclusion

Our findings provide insights into the characterization and regulatory mechanism of a novel circRNA (circPPFIA1) in LSCC. We first validated that circPPFIA1 sponges miR-340-3p and inhibits the expression of ELK1 to accelerate LSCC progression. These findings provide a theoretical basis for the possibility of circPPFIA1 as a novel target for the diagnosis and treatment of LSCC.
